# A comparative analysis on serious adverse events reported for COVID-19 vaccines in adolescents and young adults

**DOI:** 10.3389/fpubh.2023.1145645

**Published:** 2023-06-12

**Authors:** Beniamino Cappelletti-Montano, Giuseppe Demuru, Ezio Laconi, Monica Musio

**Affiliations:** ^1^Department of Mathematics and Computer Sciences, University of Cagliari, Cagliari, Italy; ^2^Department of Biomedical Sciences, University of Cagliari, Cagliari, Italy

**Keywords:** COVID-19 vaccines, Influenza vaccines, HPV vaccines, Monkeypox vaccines, VAERS, pharmacovigilance, SARS-Cov-2

## Abstract

This study aims to assess the safety profile of COVID-19 vaccines (mRNA and viral vector vaccines) in teenagers and young adults, as compared to Influenza and HPV vaccines, and to early data from Monkeypox vaccination in United States.

**Methods:**

We downloaded data from the Vaccine Adverse Event Reporting System (VAERS) and collected the following Serious Adverse Events (SAEs) reported for COVID-19, Influenza, HPV and Monkeypox vaccines: deaths, life-threatening illnesses, disabilities, hospitalizations. We restricted our analysis to the age groups 12–17 and 18–49, and to the periods December 2020 to July 2022 for COVID-19 vaccines, 2010–2019 for Influenza vaccines, 2006–2019 for HPV vaccines, June 1, 2022 to November 15, 2022 for Monkeypox vaccine. Rates were calculated in each age and sex group, based on an estimation of the number of administered doses.

**Results:**

Among adolescents the total number of reported SAEs per million doses for, respectively, COVID-19, Influenza and HPV vaccines were 60.73, 2.96, 14.62. Among young adults the reported SAEs rates for, respectively, COVID-19, Influenza, Monkeypox vaccines were 101.91, 5.35, 11.14. Overall, the rates of reported SAEs were significantly higher for COVID-19, resulting in a rate 19.60-fold higher than Influenza vaccines (95% C.I. 18.80–20.44), 4.15-fold higher than HPV vaccines (95% C.I. 3.91–4.41) and 7.89-fold higher than Monkeypox vaccine (95% C.I. 3.95–15.78). Similar trends were observed in teenagers and young adults with higher Relative Risks for male adolescents.

**Conclusion:**

The study identified a risk of SAEs following COVID-19 vaccination which was markedly higher compared to Influenza vaccination and substantially higher compared to HPV vaccination, both for teenagers and young adults, with an increased risk for the male adolescents group. Initial, early data for Monkeypox vaccination point to significantly lower rates of reported SAEs compared to those for COVID-19 vaccines. In conclusion these results stress the need of further studies to explore the bases for the above differences and the importance of accurate harm-benefit analyses, especially for adolescent males, to inform the COVID-19 vaccination campaign.

## 1. Introduction

Vaccination against COVID-19, started at the end of 2020, was a cornerstone in mitigating the harmful effects of the pandemic in the most vulnerable people.

Because of the promising initial, short-term data on effectiveness against infection (1), some governments required vaccination passports to access public offices, schools, workplaces, etc. Large segments of the population were forced to receive the vaccine, regardless of individual risk of developing a severe COVID-19 disease. However, as pointed out in Fraiman et al. (2), based on the safety data of the pivotal authorization trials of the bnt162b2 and mrna-1273 vaccines (3, 4), a risks-benefits analysis, could (have been) be useful particularly for the younger age groups [*cf.* also Bardosh et al. (5)]. In this regard, it is worth mentioning the recent decision of the Danish Health Authority, to no longer recommend vaccination to people under 50 years of age, in view of the lower severity of the Omicron variant (although children and young people who are at increased risk of a serious course of COVID-19 will continue to have the option of vaccination after individual assessment).^1^

The clinical trials required for obtaining the Emergency Authorization^2^ were statistically powered to estimate vaccine efficacy against symptomatic disease but, of course, might not have been adequate to assess safety on a large scale since in general some adverse events may be rare while vaccine trials involve a limited population. Thus the analyses of post-marketing adverse events data becomes of crucial importance. In order to monitor the safety of COVID-19 vaccines, regulatory agencies have largely used passive surveillance systems, like VAERS in United States and EudraVigilance in Europe. Such systems have several limitations, including data collection bias, reporting errors and the well-known phenomenon of underreporting (6–8). Nevertheless, such surveillance systems could play an important role in vaccination campaigns, since they serve as an early warning or signaling system for adverse events not detected during pre-market testing. Indeed, over time, through reports on VAERS and EudraVigilance, some side effects associated to COVID-19 vaccines emerged, such as venous sinus thrombosis (especially for viral vector vaccines), myocarditis/pericarditis (especially for mRNA vaccines), and others reported in the literature (9–11). While it is important to note that the adverse events reports do not necessarily indicate a cause-effect relation, rigorous analyses of these reports may provide a “warning bell” and suggest further in-depth studies to be carried out.

It was observed that the trend of VAERS reported adverse events over the years has accelerated sharply since the rollout of COVID-19 vaccines (12). Similarly, the number of hospitalizations, deaths, and, more generally, serious adverse events, as well as some very specific symptoms, have also experienced unprecedented increases since 2021.^3^ However, these pictures, while suggestive, are useless until one provides some “measuring tape.” This becomes even more important in a situation, such as the COVID-19 pandemic, in which new types of vaccines have been brought to market.

There are indeed at least two problems to be addressed. The first is that, because of the novelty of the COVID-19 vaccines (mRNA and viral-vector technologies), there were no known adverse reactions to be focused on. Secondly, the underreporting makes it not possible to compare the rate of any adverse event with the rate of the same adverse event in the general population, assuming that the latter is known. Incidentally, this kind of mistake was made by the Italian Regulatory Agency (AIFA), which compared the rate of reported deaths within 14 days after vaccination with the probability of dying from any cause in 14 days in the general Italian population [(13) p. 23].

To address the first problem, the Brighton Collaboration and the Coalition for Epidemic Preparedness Innovations partnership, Safety Platform for Emergency vACcines (SPEAC), set up and then updated a «priority list of potential adverse events of special interest (AESIs) relevant to COVID-19 vaccine trials», based, among others, on the SARS-CoV-2 specific immunopathogenesis and on the adverse events reported in prior vaccines (14). To address the second problem, a possible option is to compare the numbers of reported adverse events per million doses of COVID-19 vaccines with the same rates for other vaccines prior to the COVID-19 pandemic. This strategy rests on the assumption that the rates of underreporting do not differ significantly among different vaccines and time periods. Based on this premise, in the present study we provide a descriptive analysis of reported adverse events for COVID-19 vaccines, in the VAERS database, compared, whenever is possible, with other vaccines commonly used in the United States, such as Influenza and HPV vaccines. In our report we also included an exploratory analysis of early data from Monkepox vaccine, whose administration started in mid-2022 following the Monkeypox outbreak. We concentrated our analysis in the 12–17 and 18–49-years age groups, mainly for two reasons. Firstly, in teenagers the effects of confounding factors that can heavily impact the results of the analysis are minimized. Secondly, we aimed to contribute towards a better understanding of the risk–benefit ratio for younger age groups, and for this reason we specifically focused our analysis on the *serious* adverse events reported in VAERS database.

## 2. Materials and methods

### 2.1. Reported adverse events data

We downloaded data from VAERS website^4^ relative to the COVID-19 vaccines mrna-1273, bnt162b2 and Ad26.COV2.S (years 2020–2022 until 31 of July 2022), and Influenza (years 2010–2019), HPV (years 2006–2019) and Monkeypox (year 2022).

Data from VAERS database are collected in three CSV extension files for each year, named “VAERSData,” “VAERSSYMPTOMS” and “VAERSVAX.”

In the VAERSData-file, each report is described by 35 variables, including a VAERS_ID identifier and other information showing, among other things, the date the report was received, the patient’s State, age, sex, and a brief description of symptoms. Also, some binary variables indicating whether the patient died, had life-threatening conditions, was hospitalized or developed permanent disabilities are provided. Finally, other variables are the date of vaccination, the date of death, if any, the date of manifestation of the adverse event and other medical information. In the VAERSSYMPTOMS-file for each record we have 11 variables: in addition to the VAERS_ID, 5 of them are used to describe separately individual symptoms possibly manifested by the patient. In the VAERSVAX-file there are 8 variables including the type of vaccine administered, the corresponding pharmaceutical company, the batch and the number of doses received by the patient, and of course the identifier VAERS_ID.

We downloaded all these files, merged them, and analyzed the data by the statistical software “R.”

In this analysis we will focus on the following Serious Adverse Events (SAEs): death, permanent disability, life-threatening illness, hospitalization.^5^

We have cleaned the data by only considering reports for which each date (vaccination, death, onset of the adverse event) is:

- between 13/12/2020 and 31/07/2022 with regard to COVID-19 vaccines;
- between 01/07/2010 and 30/06/2019 with regard to Influenza vaccines;
- between 01/01/2006 and 31/12/2019 with regard to HPV vaccines; and
- between 01/06/2022 and 15/11/2022 with regard to Monkeypox vaccines.

We deleted the reports where the difference between the date of onset of the adverse event or death and the date of vaccination is negative, indicating possible errors in filing the report. We also have excluded duplicated reports.

In our analysis we deal with reported serious adverse events in the adolescent and young adults age groups. Notice that in the CDC databases considered, the former category is identified with the age group 12–17 for COVID-19 and Monkeypox, while with the age group 13–17 for Influenza and HPV vaccines. Therefore, such slight difference is present in our analysis.

### 2.2. Vaccines doses data

We downloaded the official data on administered doses of COVID-19 vaccines at the CDC Data Catalog website.^6^ Here it is possible to track the daily cumulative number of people with 1/2/3 doses in every single State (and in the global United States), classified, among other things, in the following demographic categories:

“Male(Female)_Ages_12–17_yrs”;
“Male(Female)_Ages_18–24_yrs”;
“Male(Female)_Ages_25-49_yrs.”

We then estimated the weekly number of doses administered in the two age groups of interest, 12–17 and 18–49. Namely, for each day and demographic group of interest, we calculated the total number of administered doses using the formula $3N_{3}+2\left(N_{2}-N_{3}\right)+\left(N_{1}-N_{2}\right)$ , where $N_{1},N_{2},N_{3}$ denote, the number of people with at least one, two or three doses, respectively.

The actual daily values were obtained from the cumulative daily data by subtracting the numbers obtained each day from those of the previous day; finally, we computed the weekly data by adding actual daily numbers for each week.

Unfortunately, data resolution for Influenza vaccines is not as detailed as for COVID-19. However, the weekly numbers of distributed doses are available.^7^ As Influenza vaccines have been used for several years, the number of total distributed doses can be considered a good proxy of those actually administered. Furthermore, the percentage estimates of the vaccination coverage, based on telephone interviews, are available at the CDC website: https://www.cdc.gov/flu/fluvaxview/coverage-by-season.htm. Combining these two sources we can estimate the number of yearly administered doses in each age and gender group, as reported in Tables 1, 2.

**Table 1:** Estimated influenza vaccines administered doses for the age group 13–17 (millions).

Season	Total	Males	Females
2010–2011	8.43	4.24	4.19
2011–2012	7.06	3.46	3.60
2012–2013	8.48	4.22	4.26
2013–2014	8.90	4.44	4.46
2014–2015	9.56	4.70	4.86
2015–2016	9.74	4.84	4.90
2016–2017	9.91	4.99	4.92
2017–2018	11.46	5.69	5.77
2018–2019	11.68	5.80	5.88

**Table 2:** Estimated Influenza vaccines administered doses for the age group 18–49 (millions).

Season	Total	Males	Females
2010–2011	48.1	21.5	26.6
2011–2012	39.5	17.5	22.0
2012–2013	40.5	18.1	22.4
2013–2014	41.0	17.9	23.1
2014–2015	45.1	20.1	25.0
2015–2016	44.9	19.8	25.1
2016–2017	44.6	19.5	25.1
2017–2018	42.4	18.7	23.7
2018–2019	50.9	22.2	28.7

Updated data of Jynneos, the recommended Monkeypox vaccine in United States, are available at the Monkyepox response section in the CDC website.^8^ There it is possible to download the number of weekly administered doses and of the total administered doses by age and sex. As of November 15, 2022, 1,090,222 doses were administered. Tables 3, 4 show the distribution of administered doses for different age and sex classes. Since the vast majority of doses were administered to young adult men, we restricted our exploratory analysis to men aged 18–49 years.

**Table 3:** Monkeypox vaccine doses in United States per age groups, as of November 15, 2022.

Age group	% Doses administered
0–4 years	0.03%
5–11 years	0.04%
12–17 years	0.06%
18–24 years	7.06%
25–39 years	45.58%
40–49 years	18.56%
50–64 years	22.58%
65+ years	6.10%
Unknown	0.00%

**Table 4:** Monkeypox vaccine doses in United States per sex groups, as of November 15, 2022.

Sex group	% Doses administered
Male	91.0%
Female	7.4%
Unknown	1.6%

Finally, we estimated the number of doses of HPV vaccines. The first HPV vaccine became available in the United States in late 2006 in a 3-doses schedule for girls aged 9–26 years. Routine vaccination at age 11 or 12 years has been recommended by the Advisory Committee on Immunization Practices (ACIP) since 2006 for females and since 2011 for males. Since 2014 a 2-doses schedule was recommended for girls and boys aged 9 through 14 years, and a 3-doses schedule for those at ages 15 through 26 years and for immunocompromised persons (15, 16).

The coverage among adolescent in the age class 13–17 years is quite high. In 2021 it is estimated that 78.5% and 75.4% among 13–17 girls and boys, respectively, in United States received at least 1 dose. In Table 5 we summarize the coverage in the period 2006–2019.^9^

**Table 5:** Human papillomavirus (HPV) vaccination coverage (≥ 1 dose) among adolescents 13–17 years in United States.

Year	% Females, ≥ 1 dose	% Males, ≥ 1 dose
2006	–	–
2007	25.1	–
2008	37.2	–
2009	44.3	–
2010	48.7	1.4
2011	53.0	8.3
2012	53.8	20.8
2013	57.3	34.6
2014	60.0	41.7
2015	62.8	49.8
2016	65.1	56.0
2017	68.6	62.6
2018	69.9	66.3
2019	73.2	69.8

Unfortunately, the exact number of yearly administered doses of HPV vaccines is not available. However, in the HRSA website^10^ it is possible to find the official data of cumulative distributed doses in the period 2006–2019, namely 132,062,306 doses. This number, because of the common use of HPV vaccine among adolescents, the absence of variant-adapted vaccine updates and the long period considered, can be taken as a proxy of the total amount of doses administered during the period 2006–2019. To estimate the percentage of vaccinated females and males, we combined the data of Table 5 with the United States 13–17 population in each sex group and in each year, available at the CENSUS website.^11^ We then assume that the number v(s,t) of new vaccinated adolescents in the sex class *s* ∈ {female, male} and year *t* ∈ {2006, …, 2019} can be estimated by the formula


v(s,t)=p(s,t,1)⋅N(s,t)−α(s,t)⋅v(s,t−1),

where p(s,t,1) denotes the proportion of people with at least 1 dose among the adolescents of sex *s* in the year *t*, N(s,t) the total number of adolescents of sex *s* in the year *t* and α(s,t) is the proportion of adolescents in the age class 13–17, of the sex *s* and in the year *t* − 1 who still belong to this age category in the year *t*. Finally, v(s,2006) is assumed to be 0 since the vaccination program started just at the end of 2006. According to this model, the proportion of doses administered to females can be expressed by the formula


$$\frac{1}{\sum_{t}\sum_{s}v\left(s,t\right)}\underset{t}{\sum }v\left(\mathrm{female,}t\right)$$

which gives approximately 62%. Of course, the corresponding males share is 38%.

### 2.3. Statistical analysis

Since we have available detailed data on the administered doses of COVID-19 vaccines, it is informative to calculate the weekly and cumulative rate of reported serious adverse events at each week. For this computation one should consider the lag-time between the administration of the vaccine dose and the onset of the adverse event. One possibility is to take as lag-time the median of the differences between the variables “onset date” and “vax date” in each VAERS report.

More specifically, we have computed the number of weekly deaths (per MMWR weeks) reported in the period 13/12/2020–31/07/2022. The missing values were distributed proportionally to the number of deaths in each age group and week. We have then restricted the analysis to the age categories 12–17, 18–49 and we have divided such values for the number of doses administered T0 weeks before the week of vaccination, where T0 indicates the median of the period between the vaccination and the onset week. Similar computations were done for the remaining serious adverse events categories, i.e., “Life Threatening,” “Hospital admission,” “Disability.” The corresponding graphs are available in the Section 3.

We have then calculated for COVID-19 and other vaccines the proportion of VAERS reports with at least one serious adverse event, in the two age classes considered. Formally, let


$$P_{C,a}=\frac{{\mathrm{SAE}}_{C,a}}{N_{C,a}}$$

and


$$P_{V,a}=\frac{{\mathrm{SAE}}_{V,a}}{N_{V,a}}$$

be such proportions. Here $N_{C,a}$ and $N_{V,a}$ denote the total number of administered doses and $S{\mathrm{AE}}_{C,a}$ and ${\mathrm{SAE}}_{V,a}$ denote the number of reports with at least a serious adverse event in the sex-age class age *a* for COVID-19 vaccines and the vaccine *V*, respectively. Then we compute the Relative Risk


$${\mathrm{RR}}_{V,a}=\frac{P_{C,a}}{P_{V,a}}$$

and the associated 95%—confidence interval for each age group. We remind that the standard error of the natural logarithm of the Relative Risk is


$${\hat{\sigma }}_{RR_{a}}=\sqrt{\frac{1-P_{C,a}}{{\mathrm{SAE}}_{C,a}}+\frac{1-P_{V,a}}{{\mathrm{SAE}}_{V,a}}}.$$

In Table 6 we report, for each group *a*, the values of $P_{C,a}$ , $P_{V,a}$ and ${\mathrm{RR}}_{V,a}$ for different vaccines *V*. Similar computations have also been done for reported deaths, life-threatening illnesses, disabilities, and hospitalizations. For Monkeypox vaccine, due to the relatively small window time, we only computed the Relative Risk for the totality of SAEs.

## 3. Results

Absolute numbers of reported SAEs are listed in Table 7. Concerning COVID-19 vaccination, a total of 27,559 serious reactions of 22,324 different vaccine recipients aged 12–49 years are included. As for Influenza vaccines, a total of 2,375 serious reactions of 1,943 different vaccine recipients aged 13–49 years are included. A total of 1,931 serious reactions of 1,509 different adolescents aged 13–17 years are included regarding HPV vaccinations. A total of 8 serious reactions of 6 different male adults aged 18–49 years are included regarding Monkeypox vaccinations.

**Table 7:** Total numbers of reported Serious Adverse Events (SAEs) in adolescents and young adults from VAERS data for COVID-19, Influenza, HPV and Monkeypox vaccines.

Vax type	Data collection date (dd/mm/yy)	Sex and age groups				Total
		Male adolescents	Female adolescents	Male adults	Female adults	
COVID-19	13/12/2020–31/07/2022	1,492	769	10,430	14,868	27,559
Influenza	01/07/2010–30/06/2019	117	135	732	1,391	2,375
HPV	01/01/2006–31/12/2019	255	1,676	–	–	1,931
Monkeypox	01/06/2022–15/11/2022	–	–	8	–	8

The distribution of the different SAEs categories (death, life-threatening, disabilities, and hospitalization), differentiated by age group and gender, are provided in Table 8, respectively, for COVID-19, Influenza, HPV and Monkeypox vaccines. Such distributions are similar among COVID-19, Influenza and HPV vaccines, with most SAEs being “hospitalizations,” and less than 5% being “deaths.” The limited number of reported SAEs for Monkeypox vaccine makes immature and not reliable the corresponding analysis for this vaccine.

Table 9 shows the most frequent and relevant symptoms included in the SAEs reports, for each age and gender group and for each vaccine type. Note that each report may include many symptoms and may include no symptom as well. Because of the immaturity of data for Monkeypox vaccine, we have not considered it in this analysis. Moreover, due to the limited number of reported symptoms for Influenza vaccines among adolescents, we have merged the males and females’ data for this age group. Symptoms with percentages less than 3% over the total number of SAEs reports have not been included. We have excluded the most common mild symptoms like injection site pain, fever, etc. since the focus of this paper is the analysis of serious adverse events.

According to our analysis, most of the reported symptoms following COVID-19 vaccination relate to heart problems. This phenomenon is particularly pronounced for male adolescents, among whom more than one third of the SAEs reports include *troponin increased* and *chest pain* as reported symptoms. Further symptoms with high prevalence following COVID-19 vaccination are *myocarditis*, *electrocardiogram abnormal*, *dyspnea*, *thrombosis*. This appears in line with the well-documented findings that male young adults are at higher risk of developing heart inflammation diseases [see for instance (17)].

Symptoms following Influenza and HPV vaccination appear to be more various, with a prevalence of *dyspnea*, *Guillain-Barré syndrome*, *paraesthesia*, *pain in extremity* for Influenza vaccines, and *syncope / loss of consciousness*, *convulsion*, *dyspnea, dizziness* for HPV vaccines, although they remain numerically limited especially for Influenza vaccines.

We point out that although Influenza and HPV data cover a significantly longer time interval than COVID-19 ones (10 years for Influenza and 13 years for HPV), in each age group the absolute number of reports, and hence also the number of reported symptoms, is generally significantly lower. In fact, the Relative Risk (RR) for the most frequent symptoms following Influenza and HPV vaccinations still have a RR > 1 for COVID-19 vaccines compared to Influenza and HPV ones, while those most frequent for COVID-19 vaccines have a very high Relative Risks for the COVID-19 vaccines arm compared to Influenza and HPV ones, with a peak for the symptom “troponin increased” in the adolescent group of RR > 500 for COVID-19 compared to Influenza vaccines, and RR > 300 for COVID-19 compared to HPV vaccines.

**Figure 1:**
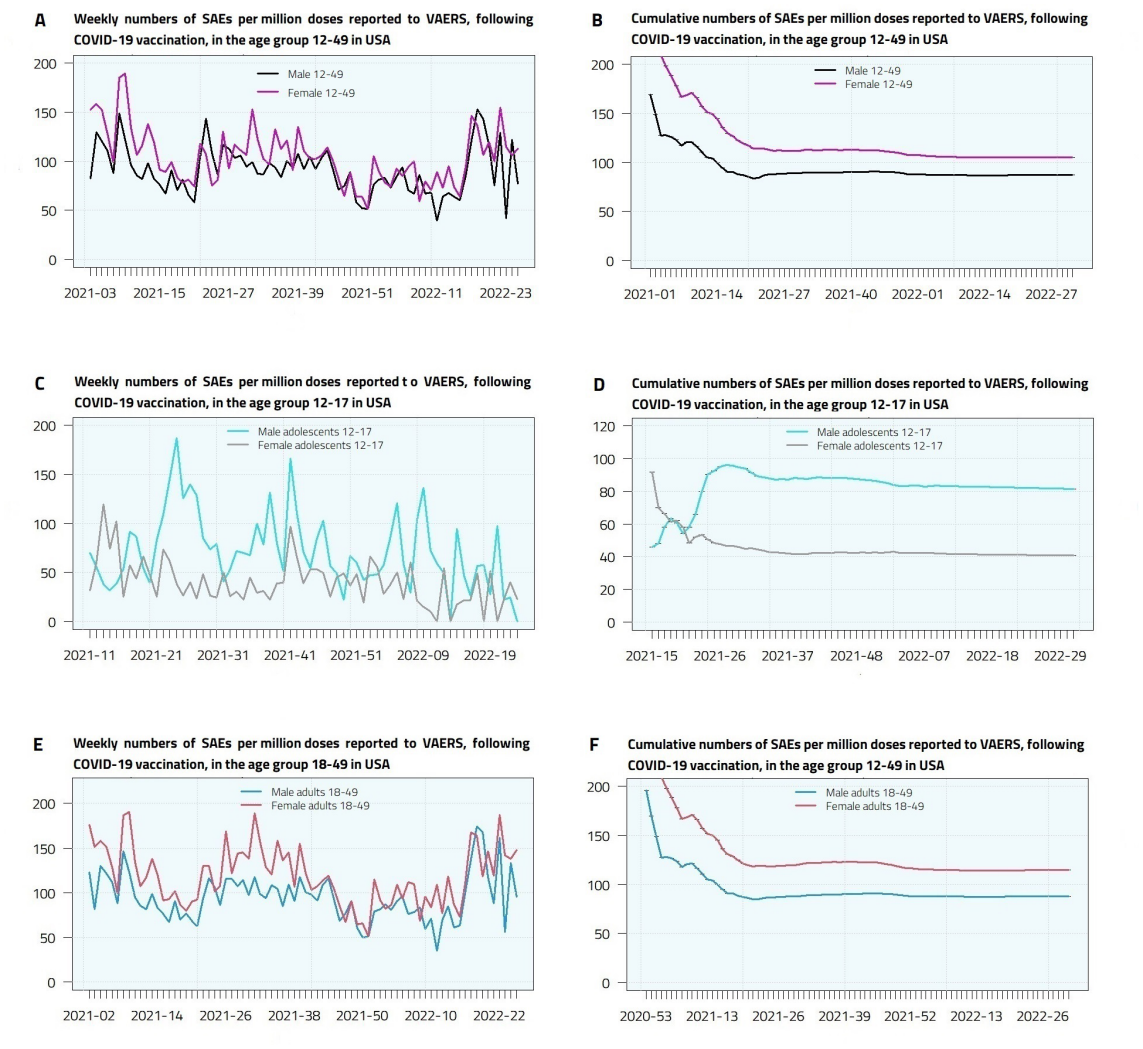
Weekly **(A,C,E)** and cumulative **(B,D,F)** Serious Adverse Events (deaths, disabilities, life-threatening illnesses, and hospitalizations) per million doses reported to VAERS, following COVID-19 vaccination, in male and female adolescents and young adults in United States.

The time series of the total (12–49) number of SAEs per million doses reported for COVID-19 vaccines show a similar pattern for male and female, except for the last period considered (week 2022–19 and later). A larger variation over time is present if we differentiate by age group, especially in the age group 12–17 (Figures 1A,C,E), with higher peaks in males in weeks 2021–24, 2021–41 and 2022–09. The different behavior between males and females is confirmed by a statistical test for comparing the similarities between the two-time series of weekly proportions (*p*-value < 0.0001). A different pattern is observed in the 18–49 age group, where there is a prevalence of female SAEs and peaks are present in weeks 2021–08, 2021–31 and 2022–22. As expected, the cumulative plots (Figures 1B,D,F) show an asymptotic trend, reaching the values of 87.04 (males 12–49), 105.30 (females 12–49), 87.92 (male adults), 114.71 (female adults), 81.35 (male adolescents), 40.71 (female adolescents).

**Figure 2:**
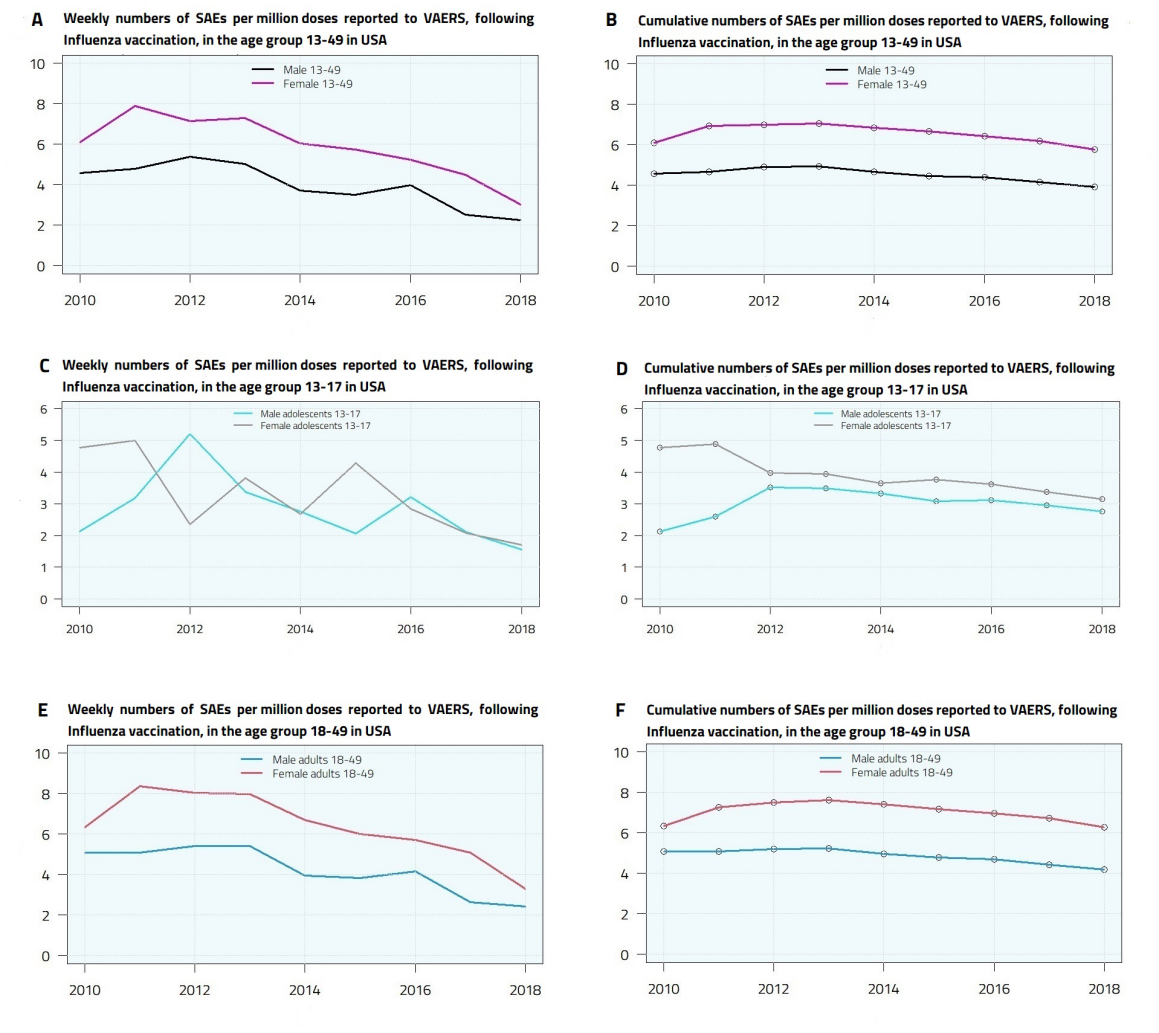
Yearly **(A,C,E)** and cumulative **(B,D,F)** Serious Adverse Events (deaths, disabilities, life-threatening illnesses, and hospitalizations) per million doses reported to VAERS, following Influenza vaccination, in male and female adolescents and young adults in United States.

For Influenza vaccines, weekly administered doses data are not available, so it is more appropriate to consider the rate of reported SAEs by season. The total number of reported SAEs is shown in Table 10. In Figure 2 we have plotted the trend of yearly and cumulative reported SAEs numbers per million doses. The cumulative plots show a slightly decreasing behavior, reaching the values of 3.90 (male 13–49), 5.77 (female 13–49), 4.18 (male adults), 6.27 (female adults), 2.76 (male adolescents), 3.15 (female adolescents).

**Table 10:** Total number of reported SAEs for Influenza vaccines by season, sex and age groups.

Season	Males 13–17	Females 13–17	Males 18–49	Females 18–49
2010–2011	9	20	109	168
2011–2012	11	18	89	184
2012–2013	22	10	98	180
2013–2014	15	17	97	184
2014–2015	13	13	79	167
2015–2016	10	21	76	151
2016–2017	16	14	81	143
2017–2018	12	12	49	120
2018–2019	9	10	54	94

**Table 8:** Distribution of the different SAEs types for different age and sex groups.

Vaccine	Age and sex group	Deaths	Life threatening	Disabilities	Hospitalizations

COVID-19	*All 12–49*	*0.0378*	*0.1710*	*0.2120*	*0.5792*
	Males 12–49	0.0509	0.1681	0.1845	0.5965
	Females 12–49	0.0279	0.1733	0.2329	0.5659
	*Adolescents 12–17*	*0.0198*	*0.1435*	*0.0885*	*0.7481*
	Males 12–17	0.0134	0.1405	0.0649	0.7813
	Females 12–17	0.0322	0.1495	0.1340	0.6843
	*Adults 18–49*	*0.0394*	*0.1735*	*0.2230*	*0.5640*
	Males 18–49	0.0563	0.1722	0.2015	0.5700
	Females 18–49	0.0277	0.1745	0.2381	0.5597

Influenza	*All 13–49*	*0.0298*	*0.1942*	*0.1909*	*0.5851*
	Males, 13–49	0.0397	0.1857	0.1647	0.6098
	Females, 13–49	0.0242	0.1989	0.2055	0.5713
	*Adolescents 13–17*	*0.0431*	*0.2000*	*0.1294*	*0.6274*
	Males 13–17	0.0254	0.1949	0.1441	0.6356
	Females 13–17	0.0584	0.2044	0.1168	0.6204
	*Adults 18–49*	*0.0286*	*0.1936*	*0.1983*	*0.5795*
	Males 18–49	0.0420	0.1843	0.1680	0.6057
	Females 18–49	0.0215	0.1986	0.2143	0.5656

HPV	*All 13–17*	*0.0529*	*0.1614*	*0.2859*	*0.4997*
	Males 13–17	0.0465	0.1744	0.1938	0.5853
	Females 13–17	0.0539	0.1594	0.2999	0.4867

Monkeypox	*Males 18–49*	*0.1250*	*0.2500*	*0*	*0.6250*

**Table 9:** Most frequent symptoms included in SAEs reports for different age and sex groups for COVID-19, Influenza and HPV vaccines.

		COVID-19 vaccines	Influenza vaccines	HPV vaccines
Adolescents, males	SAEs reports	*1,257*	*203*	*201*
	Most frequent relevant symptoms	Troponin increased (34.3%) Chest pain (32.6%) Myocarditis (23.8%) Electrocardiogram abnormal (16.8%) Dyspnea (5.9%) C-reactive protein increased (5.6%) Pericarditis (3.8%) Appendicitis (3.2%) Brain natriuretic peptide increased (3.0%)	Dyspnea (6.9%) Guillain-Barré syndrome (5.9%) Immunoglobulin therapy (5.9%) Convulsion (4.4%) Chest pain (3.9%) Gait disturbance (3.9%) Platelet count decreased (4.0%) Dizziness (3.5%) Pain in extremity (3.5%)	Syncope/loss of consciousness (6.5%) Immunoglobulin therapy (5.5%) Convulsion (4.5%) Dyspnea (4.5%) Chest pain (4.0%) Platelet count decreased (4.0%) Seizure (4.0%) Type I diabetes mellitus (4.0%)
Adolescents, females	SAEs reports	*638*		*1,308*
	Most frequent relevant symptoms	Chest pain (8.8%) Dyspnea (5.9%) Troponin increased (5.6%) Seizure (4.7%) Myocarditis (4.4%) Syncope / loss of consciousness (3.6%) C-reactive protein increased (3.4%) Dizziness (3.4%)		Syncope / loss of consciousness (9.9%) Dizziness (7.6%) Convulsion (6.4%) Dyspnea (4.3%) Pain in extremity (4.0%)
Young adults, males	SAEs reports	*8,248*	*590*	-
	Most frequent relevant symptoms	Chest pain (12.9%) Dyspnea (9.5%) Myocarditis (7.1%) Troponin increased (5.7%) Thrombosis (4.8%) Myocardial infarction (4.0%) Pulmonary embolism (3.8%) Tinnitus (3.3%) Dizziness (3.1%) Electrocardiogram abnormal (3.0%)	Paraesthesia (8.3%) Guillain-Barré syndrome (7.6%) Pain in extremity (7.1%) Dyspnea (4.9%) Dizziness (4.7%) Paralysis (4.4%) Chest pain (4.2%) Immunoglobulin therapy (3.5%)	-
Young adults, females	SAEs reports	*12,181*	*1,152*	-
	Most frequent relevant symptoms	Dyspnea (9.5%) Chest pain (6.4%) Dizziness (5.0%) Thrombosis (4.5%) Pain in extremity (4.2%) Pulmonary embolism (4.0%)	Guillain-Barré syndrome (10.8%) Dyspnea (10.0%) Paraesthesia (7.7%) Pain in extremity (5.6%) Dizziness (4.3%) Immunoglobulin therapy (4.0%)	-

Percentages are referred to the total number of distinct reports in each category (each report may contain many symptoms). Symptoms with percentages less than 3% are not included.

**Table 6:** Reported SAEs per million doses for COVID-19 vaccines, Influenza, HPV, Monkeypox vaccines and corresponding relative risks (95% C.I.)

	Total reported SAEs rate				Relative risk		
	COVID-19	Influenza	HPV	Monkeypox	RR*_COVID-19, Influenza_*	RR*_COVID-19, HPV_*	RR*_COVID-19, Monkeypox_*
*Overall*	*96.54*	*4.93*	*–*	*–*	*19.60 (18.80, 20.44)*	*–*	–
Males	87.04	3.90	–	–	22.32 (20.82, 23.93)	–	–
Females	105.30	5.77	–	–	18.25 (17.32, 19.24)	–	–
*Adolescents*	*60.73*	*2.96*	*14.62*	*–*	*20.54 (18.03, 23.40)*	*4.15 (3.91, 4.41)*	*–*
Male adolescents	81.35	2.76	5.07	–	29.47 (24.41, 35.57)	16.05 (14.05, 18.33)	–
Female adolescents	40.71	3.15	20.50	–	12.92 (10.76, 15.50)	1.99 (1.82, 2.16)	–
*Adults*	*101.91*	*5.35*	*–*	*–*	*19.05 (18.23, 19.92)*	*–*	*–*
Male adults	87.92	4.18	–	11.14	21.05 (19.54, 22.70)	–	7.89 (3.95, 15.78)
Female adults	114.71	6.27	–	–	18.28 (17.3, 19.32)	–	–

**Table 11:** Reported SAEs resulting in Death per million doses for COVID-19 vaccines, Influenza and HPV vaccines and corresponding relative risks (95% C.I.)

	Reported deaths rate			Relative risk	
	COVID-19	Influenza	HPV	RR*_COVID-19, Influenza_*	RR*_COVID-19, HPV_*
*Overall*	*3.61*	*0.15*	*-*	*24.88 (19.53, 31.69)*	*-*
Males	4.40	0.15	-	29.04 (20.46, 41.23)	-
Females	2.88	0.14	-	20.61 (14.73, 28.83)	-
*Adolescents*	*1.15*	*0.12*	*0.73*	*9.84 (4.95, 19.59)*	*1.59 (1.11, 2.28)*
Male adolescents	1.04	0.07	0.22	14.64 (4.33, 49.46)	4.74 (2.25, 9.96)
Female adolescents	1.27	0.16	1.04	7.78 (3.35, 18.05)	1.22 (0.78, 1.92)
*Adults*	*3.98*	*0.15*	*-*	*26.33 (20.29, 34.17)*	*-*
Male adults	4.92	0.17	-	28.77 (19.93, 41.52)	-
Female adults	3.12	0.14	-	23.03 (15.90, 33.38)	-

**Table 12:** Reported SAEs resulting in Life-threatening illnesses per million doses for COVID-19 vaccines, Influenza and HPV vaccines and corresponding relative risks (95% C.I.)

	Reported life-threatening SAEs rate			Relative risk	
	COVID-19	Influenza	HPV	RR*_COVID-19, Influenza_*	RR*_COVID-19, HPV_*
*Overall*	*16.49*	*0.92*	*-*	*17.91 (16.25, 19.74)*	*-*
Males	14.62	0.72	-	20.40 (17.33, 24.00)	-
Females	18.22	1.15	-	15.85 (14.08, 17.85)	-
*Adolescents*	*8.62*	*0.56*	*2.36*	*15.31 (11.30, 20.73)*	*3.65 (3.12, 4.26)*
Male adolescents	11.40	0.52	0.87	21.95 (14.15, 34.06)	13.03 (9.41, 18.04)
Female adolescents	5.93	0.65	3.28	9.07 (6.00, 13.73)	1.81 (1.45, 2.25)
*Adults*	*17.67*	*1.03*	*-*	*17.11 (15.46, 18.93)*	*-*
Male adults	15.11	0.76	-	19.77 (16.59, 23.57)	-
Female adults	20.01	1.24	-	16.07 (14.19, 18.19)	-

**Table 13:** Reported SAEs resulting in Disabilities per million doses for COVID-19 vaccines, Influenza and HPV vaccines and corresponding relative risks (95% C.I.)

	Reported SAEs resulting in disabilities rate			Relative risk	
	COVID-19	Influenza	HPV	RR*_COVID-19, Influenza_*	RR*_COVID-19, HPV_*
*Overall*	*20.52*	*0.94*	*–*	*21.89 (19.89, 24.09)*	*–*
Males	16.10	0.64	–	25.21 (21.24, 29.92)	–
Females	24.59	1.18	–	20.71 (18.52, 23.33)	–
*Adolescents*	*5.35*	*0.38*	*4.19*	*14.23 (9.80, 20.68)*	*1.28 (1.09, 1.50)*
Male adolescents	5.29	0.40	0.99	13.19 (7.88, 22.07)	5.32 (3.78, 7.49)
Female adolescents	5.40	0.35	6.15	15.42 (8.97, 26.52)	0.88 (0.71, 1.09)
*Adults*	*22.79*	*1.06*	*–*	*21.54 (19.51, 23.79)*	*–*
Male adults	17.77	0.70	–	25.53 (21.27, 30.65)	–
Female adults	27.39	1.34	–	20.38 (18.11, 22.93)	–

**Table 14:** Reported SAEs resulting in Hospitalizations per million doses for COVID-19 vaccines, Influenza and HPV vaccines and corresponding relative risks (95% C.I.)

	Reported hospitalizations rate			Relative risk	
	COVID-19	Influenza	HPV	RR*_COVID-19, Influenza_*	RR*_COVID-19, HPV_*
*Overall*	*55.92*	*2.89*	*–*	*19.36 (18.33, 20.45)*	*–*
Males	51.92	2.39	–	21.69 (19.85, 23.71)	–
Females	59.61	3.30	–	18.08 (16.87, 19.39)	–
*Adolescents*	*45.61*	*1.88*	*7.35*	*24.29 (20.66, 28.57)*	*6.21 (5.74, 6.72)*
Male adolescents	63.63	1.77	2.98	35.96 (28.47, 45.41)	21.34 (18.01, 25.30)
Female adolescents	28.11	1.98	10.03	14.17 (11.27, 17.81)	2.80 (2.51, 3.13)
*Adults*	*57.47*	*3.11*	*–*	*18.50 (17.46, 19.61)*	*–*
Male adults	50.11	2.54	–	19.70 (17.90, 21.69)	–
Female adults	64.20	3.55	–	18.09 (16.81, 19.46)	–

Official weekly/yearly numbers of administered doses are not available for HPV vaccines, and therefore similar plots could not be derived. On the other hand, while data are available for Monkeypox vaccines, numbers are still insufficient for a temporal analysis, since the vaccination program started a few months before the completion of the present study. However, as an exploratory analysis, we can consider the cumulative, total rate of reported SAEs for the computation of the Relative Risk.

As for the RR in the comparison between COVID-19, Influenza, HPV and Monkeypox vaccines, the results are collected in Tables 6, 11–14.

The RR points to a marked increase in reported SAEs rate for COVID-19 vaccines with respect to the other considered vaccines. Overall, the increase is about 20-folds compared to Influenza vaccines, 4-fold compared to HPV vaccines and 8-fold compared to Monkeypox vaccine.

**Figure 3:**
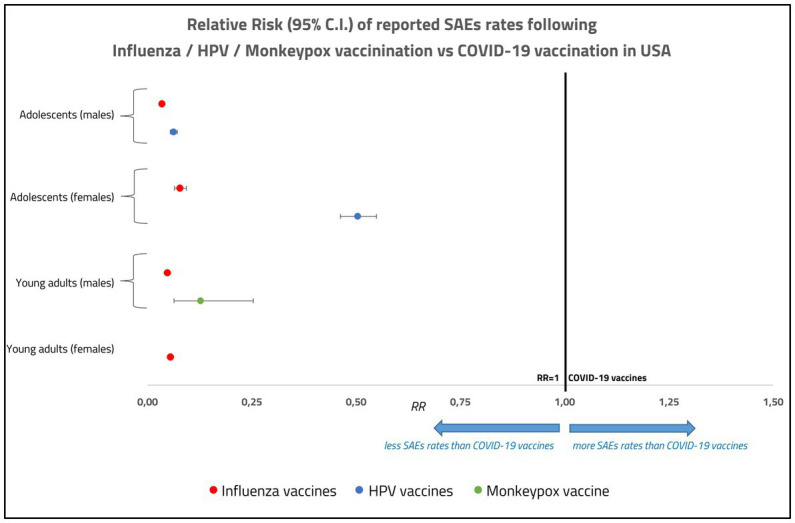
Relative risk of reported serious adverse events (deaths, disabilities, life-threatening illnesses, and hospitalizations) for Influenza, HPV and Monkeypox vaccines with respect to COVID-19 vaccines, in male and female adolescents and young adults in United States, with the corresponding confidence intervals.

When we conducted a separate analysis by age and sex groups, we identified a clear excess of reported SAEs rate in the group of male adolescents. This is illustrated in Figure 3, where we have reported the RR of SAEs for each age category and sex considering COVID-19 vaccines as reference.

## 4. Discussion

The main finding derived from our analysis is that the rate of SAEs following COVID-19 vaccination during the 2021–2022 campaign in the United States is about 20 times higher than that observed for Influenza vaccines in the years 2010–2019. Such increase is consistent, with some variability, across two age groups (teenagers and young adults), female or male sex and different types of SAEs (death, disabilities, hospitalization, life-threatening). Furthermore, higher rates of SAEs were also recorded for COVID-19 vaccines in comparison with those associated with HPV vaccination, albeit differences varied according to sex and type of SAEs (Tables 6, 11–14). Finally, a sizeable increase in the rate of SAEs was also seen when young male adults receiving COVID-19 vaccines were compared with individuals of the same sex and age range offered the newly introduced Monkeypox vaccination. Our results are in line with and extend those of Montano, who reported a higher relative risk of serious adverse reactions (including allergy, arrhythmia, cardiovascular events, hemorrhage, thrombosis, and others) in COVID-19 vs. Influenza vaccinees, in both US and Europe in the years 2020–2021 (18). In that study the focus was a comparison between the older adults (≥ 65 years) and people aged 18–64 years, and the relative risk estimates of adverse reactions were classified according to Common Toxicity Criteria (CTC).

More recent comparative analyses of VAERS identified increased risk for hearing disorders (19), new-onset seizures (20) and oral adverse events (21) following administration of COVID-19 vaccines compared to Influenza vaccination.

The reasons for the striking differences observed in SAEs following administration of COVID-19 vaccines are presently unknown and require timely attention by public health authorities and careful investigation by the research community. The first possibility to be considered is the existence of any difference in sensitivity of the passive reporting systems. In fact, given that data analyzed rely on passive pharmacovigilance, one cannot rule out a higher level of awareness for COVID-19 vaccines-related SAEs as opposed to those induced by Influenza and/or HPV vaccines. This argument becomes more pertinent since data on COVID-19, Influenza and HPV vaccines SAEs refer to different time periods, and the pandemic could have lowered the threshold of sensitivity towards reporting adverse reactions. However, in our view this possibility is unlikely to justify the very large difference in the rate of reported SAEs found in our study, on a series of accounts. COVID-19 vaccine-related SAEs were about 8 times more frequent (on average) compared to those associated with Monkeypox vaccination, the latter being performed during the COVID-19 pandemic. In addition, the study by Montano referred to above (18) compared rates of SAEs for COVID-19 vs. Influenza vaccines that were both administered during the pandemic, and yet a large difference in the rate of SAEs was found, even higher than that of our report. Lastly, the level of awareness is unlikely to play a major role when dealing with adverse reactions with relevant impact on health, such as those considered in our analysis. Thus, varying levels of awareness does not stand as a major explanatory factor of the differences in frequencies of SAEs observed for COVID-19 vaccines, and alternative hypotheses must be proposed and possibly tested.

On the other hand, one may argue that the reported SAEs following vaccination against SARS-CoV-2 could also likely be caused by the various invasive preventive measures that were unique for the pandemic time period (e.g., lockdown, social distancing, school closures, etc.), and not by the vaccines. However, in (22) it is shown that the overall AEFI reporting rate, in the Australian passive pharmacovigilance system, did not increase in 2020 with respect to previous years, despite the very rigorous and invasive preventive measures adopted in Australia. The same behavior (even a slight decline) has been reported for HPV and Influenza vaccines.

Vaccines administered in the United States were based on mRNA delivered through liposomal particles (Pfizer-BioNTech and Moderna) or, only for adults 18 years and older, DNA inserted into a viral vector (J&J/Janssen). The latter account for only less than 3% of the total number of COVID-19 vaccines doses administered in USA.^12^ Neither vaccination strategy had ever been used before on such a large scale in the human population, which makes it particularly important to monitor their efficacy and safety over time. The sizeable difference in SAEs could be hypothetically attributable to either differences in the vaccine platform (influenza vaccines were mostly based on inactivated virus and HPV on viral protein particles derived from the capsid) and/or its encoded product, the spike protein. It is difficult and it would be premature at this point to speculate on specific possibilities in any detail. As discussed by Montano (18), nucleic-acid platforms for vaccination can elicit sustained immune reactions via activation of pathways related to Toll-like receptors, interleukins, tumor necrosis factors, interferons, and others (23–25). However, numerous studies have explored the multifaceted effects exerted by the spike protein encoded by SARS-CoV-2 and the COVID-19 vaccines. For example, cross-reactivity has been described between the antibodies against the spike protein and self-antigens (26, 27). This has led to the proposal of the “spike hypothesis” to account for at least some of the adverse reactions associated with vaccination (28). Furthermore, autoimmunity has been proposed as a potential risk factor for SAEs following COVID-19 vaccination [see for instance (17)]. More studies will be necessary to examine alternative hypotheses and compare adverse events elicited using different vaccine platforms (e.g., mRNA vs. protein subunit vaccines) (29). What stands out is that COVID-19 vaccines considered in the present report are associated with a more vigorous involvement of the immune response, as suggested by Angeli et al. (30), leading to increased risk of SAEs compared to other vaccines.

An additional result that emerges from our analysis is that young adult females report a higher frequency of SAEs compared to age-matched males. This is in line with previous reports in the literature (31, 32), and highlights the fact that females have a greater innate and adaptive immune response to both infection and vaccines (33), thus reinforcing the contention that SAEs are indeed causally related to the level of immune reaction to the vaccine. However, what seems to be a peculiarity of COVID-19 vaccines is that in the adolescents subgroups males show a markedly higher SAEs rate than females. Furthermore, both in the 12–17 and in the 18–49 age groups, the Risk Ratio in the males group with respect to Influenza and HPV vaccines is significantly higher than in the females group, suggesting the presence of additional, not yet well-understood mechanisms underlying the occurrence of physiopathological processes following COVID-19 vaccination.

In summary, our findings point to a remarkable difference in the rate of SAEs associated with COVID-19 vaccines administered in the United States compared to those reported for Influenza, HPV or Monkeypox vaccination. Despite the intrinsic limitations of the analysis, related to the spontaneous nature of reports, these data require appropriate consideration, and more studies are needed to explore the basis for these differences.

## Data availability statement

VAERS data are publicly available as ZIP files for each reporting year at https://vaers.hhs.gov/data/datasets.html. Data on COVID-19 vaccination in USA by age and sex are available at https://data.cdc.gov/Vaccinations/COVID-19-Vaccination-Age-and-Sex-Trends-in-the-Uni/5i5k-6cmh. Data on cumulative doses of Influenza vaccines in USA are available at https://data.cdc.gov/Vaccinations/Weekly-Cumulative-Doses-in-Millions-of-Influenza-V/k87d-gv3u. Data on Influenza and HPV vaccination coverage in USA are available, respectively, at https://www.cdc.gov/flu/fluvaxview/coverage-by-season.htm and https://data.cdc.gov/Teen-Vaccinations/Vaccination-Coverage-among-Adolescents-13-17-Years/ee48-w5t6. Data on Monkeypox vaccination in USA are available at https://www.cdc.gov/poxvirus/monkeypox/response/2022/vaccines_data.html. Data on US population by age groups are available at https://data.census.gov/cedsci/.

## Author contributions

BC-M, EL, and MM: conceptualization, methodology and conception of the research hypotheses. GD: preparation of the data. BC-M, GD, and MM: statistical analysis of the data. EL: interpretation and medical conclusions. BC-M, GD, EL, and MM: reviewing and editing of the manuscript. All authors contributed to the article and approved the submitted version.

## Funding

This research was partially supported by the project FdS 2020 funded by Fondazione di Sardegna.

## Conflict of interest

The authors declare that the research was conducted in the absence of any commercial or financial relationships that could be construed as a potential conflict of interest.

## Publisher’s note

All claims expressed in this article are solely those of the authors and do not necessarily represent those of their affiliated organizations, or those of the publisher, the editors and the reviewers. Any product that may be evaluated in this article, or claim that may be made by its manufacturer, is not guaranteed or endorsed by the publisher.

## Footnotes

^1^https://www.sst.dk/en/english/corona-eng/vaccination-against-covid-19

^2^We remind that an Emergency Use Authorization (EUA) in the United States is an authorization granted to the Food and Drug Administration to allow the use of a drug prior to approval. As stated by the FDA’s guidance for submitting an EUA (available at: https://www.fda.gov/regulatory-information/search-fda-guidance-documents/emergency-use-authorization-medical-products-and-related-authorities), it is recommended that a request for an EUA include a well-organized summary of the available scientific evidence regarding the product’s safety and effectiveness, risks (including an adverse event profile) and benefits, and any available, approved alternatives to the product. For an unapproved product, EUA conditions for monitoring and reporting of adverse events are required to the extent practicable given the circumstances of the emergency. Conditions may be placed to enable the collection and analysis of information on the safety and effectiveness of the EUA product during the period when the authorization is in effect and for a reasonable time following such period. The primary focus of adverse event-related conditions is capturing serious adverse events and applying appropriate mechanism(s) for the collection of follow-up clinical information. Some reporting may be directed to predefined mechanisms to capture adverse event data [e.g., FDA’s Safety Information and Adverse Event Reporting System (MedWatch) or Vaccine Adverse Event Reporting System (VAERS)].

^3^See, for instance, https://www.openvaers.com/covid-data/mortality

^4^https://vaers.hhs.gov/data/datasets.html

^5^https://www.cdc.gov/vaccinesafety/pdf/vaers_factsheet1.pdf

^6^https://data.cdc.gov/Vaccinations/COVID-19-Vaccination-Age-and-Sex-Trends-in-the-Uni/5i5k-6cmh

^7^https://data.cdc.gov/Vaccinations/Weekly-Cumulative-Doses-in-Millions-of-Influenza-V/k87d-gv3u

^8^https://www.cdc.gov/poxvirus/monkeypox/response/2022/vaccines_data.html

^9^https://data.cdc.gov/Teen-Vaccinations/Vaccination-Coverage-among-Adolescents-13-17-Years/ee48-w5t6

^10^https://www.hrsa.gov/sites/default/files/hrsa/vicp/vicp-stats-07-01-22.pdf

^11^https://data.census.gov/cedsci

^12^https://covid.cdc.gov/covid-data-tracker/#vaccinations_vacc-total-admin-count-total
